# Learning from the COVID-19 Pandemic—Through Sharing and Collaboration

**DOI:** 10.3390/v17121633

**Published:** 2025-12-17

**Authors:** Julian W. Tang

**Affiliations:** Clinical Microbiology, University Hospitals of Leicester NHS Trust, 5/F Sandringham Building, Leicester Royal Infirmary, Infirmary Square, Leicester LE1 5WW, UK; jwtang49@hotmail.com; Tel.: +44-116-258-6516/3574; Fax: +44-1162551949

During the COVID-19 pandemic (which some argue is still ongoing), I led three Special Issues (SIs) that were designed to cover the talking points that arose amongst many practitioners and researchers: (1) How can we maintain our usual diagnostic services in the face of an ongoing pandemic? [1] (2) Where might the next pandemic virus come from and how might we first detect it? [2] (3) How has the pandemic changed the landscape of other viruses that affect human health? [3]. The papers that were published were broadly related to the main them of each SI, though some leeway was allowed.

In the first SI, *Diagnostic Virology during the COVID-19 Pandemic—Business as Usual,* my intention was to look at how ‘non-SARS-CoV-2’ testing was being maintained despite all the pandemic-related testing that was ongoing, so none of the papers focused on SARS-CoV-2 testing directly. The papers were selected to represent a general cross-section of what diagnostic laboratories normally do in non-pandemic times, and in some cases, how they maintained such services during the pandemic. These included assay evaluations [4,5], outbreak investigations [6,7,8], routine diagnostics and assay development [9,10,11], epidemiology and surveillance testing [12,13,14]. From these studies, it is clear that researchers and their laboratories were still managing to stay active and work on non-SARS-CoV-2 projects, demonstrating that they had some capacity to spare—which bodes well for any future pandemic.

The second SI, *Animal and Human Respiratory Viruses—Causes of the Next Pandemic*, arose from the fervent speculation around the WHO (World Health Organization) visit in early 2021 to Wuhan, China, to look for possible origins of SARS-CoV-2 [15]. The debate is still ongoing (and may never be fully resolved) about whether the virus was of natural origins, or whether it had been produced in a laboratory experiment and released by accident [16]. This SI featured papers examining potential pandemic threats from viruses originating in pigs (swine influenza) [17] and birds (avian influenza) [18,19,20], as well as the detection and surveillance of various human viruses, including RSV (respiratory syncytial virus) [21,22], influenza [23], and coronaviruses [24,25,26]. Again, whilst SARS-CoV-2 and coronaviruses were the focus of many studies, this also demonstrates that researchers were still able study the behaviour of other respiratory viruses that were still potential pandemic candidates.

Finally, in the third SI, *Impact of Pandemic Measures on the Epidemiology and Seasonality of Other Viruses*, as pandemic restrictions in most countries started to ease, researchers were asking the following question: how did the pandemic and its related restrictions affect the seasonality and behaviour of other respiratory viruses? This issue was perhaps most prominently highlighted with the global outbreaks of acute hepatitis in children, many of whom had been infected with adenoviruses [27]. Although none of the contributions to this SI included adenovirus, changes in the epidemiology of seasonal influenza [28,29,30], RSV [31,32,33], and other respiratory viruses [34,35,36] were investigated. Some studies compared surveillance data from before and after the main pandemic period; others examined the viral epidemiology from just before or since the onset of the pandemic. The various findings were likely related to the study population’s prior immunity to these seasonal viruses, which determined how they responded when these viruses returned after pandemic restrictions were lifted.

I would like to sincerely thank all the authors that contributed to these SIs, especially during such a busy time, when the demands on their time were likely multiple and unrelenting.

## References

[1] Tang J.W. Viruses (MDPI). Diagnostic Virology during the COVID-19 Pandemic—Business as Usual. https://www.mdpi.com/journal/viruses/special_issues/Diagnostic_COVID.

[2] Tang J.W. Viruses (MDPI). Animal and Human Respiratory Viruses—Causes of the Next Pandemic?. https://www.mdpi.com/journal/viruses/special_issues/24M56QOXN5.

[3] Tang J.W. Impact of Pandemic Measures on the Epidemiology and Seasonality of Other Viruses. https://www.mdpi.com/journal/viruses/special_issues/UV1ZZ172A7.

[4] Jevšnik Virant M., Uršič T., Kogoj R., Korva M., Petrovec M., Avšič-Županc T. (2022). Evaluation of Two Broadly Used Commercial Methods for Detection of Respiratory Viruses with a Recently Added New Target for Detection of SARS-CoV-2. Viruses.

[5] Herrera B.B., Mayoral R., Brites C. (2022). Development and Validation of a Rapid Screening Test for HTLV-I IgG Antibodies. Viruses.

[6] Dilcher M., Howard J.C., Dalton S.C., Anderson T., Clinghan R.T., Werno A.M. (2022). Clinical, Laboratory, and Molecular Epidemiology of an Outbreak of Aseptic Meningitis Due to a Triple-Recombinant Echovirus in Ashburton, New Zealand. Viruses.

[7] Howard-Jones A.R., Pham D., Jeoffreys N., Eden J.S., Hueston L., Kesson A.M., Nagendra V., Samarasekara H., Newton P., Chen S.C. (2022). Emerging Genotype IV Japanese Encephalitis Virus Outbreak in New South Wales, Australia. Viruses.

[8] Lindblad N., Hänninen T., Valtonen M., Heinonen O.J., Waris M., Ruuskanen O. (2022). Influenza A Outbreaks in Two Professional Ice Hockey Teams during COVID-19 Epidemic. Viruses.

[9] Bird P.W., Taylor G., Cafferata J., Gardener J., McMurray C.L., Fletcher O., Toovey O.T.R., Holmes C.W., Tang J.W. (2022). Performing under Pressure: Insights into the Diagnostic Testing Burden at a UK National Health Service Clinical Virology Laboratory during the SARS-CoV-2 Pandemic. Viruses.

[10] Kitai Y., Sato K., Shirato K., Ohmiya S., Watanabe O., Kisu T., Ota R., Takeda M., Kawakami K., Nishimura H. (2022). Variation in Thermal Stability among Respiratory Syncytial Virus Clinical Isolates under Non-Freezing Conditions. Viruses.

[11] Teo C.H.Y., Norhisham N.H.B., Lee O.F., Png S., Chai C.N., Yan G., Tang J.W., Lee C.K. (2022). Towards Next-Generation Sequencing for HIV-1 Drug Resistance Testing in a Clinical Setting. Viruses.

[12] Chon I., Saito R., Kyaw Y., Aye M.M., Setk S., Phyu W.W., Wagatsuma K., Li J., Sun Y., Otoguro T. (2023). Whole-Genome Analysis of Influenza A(H3N2) and B/Victoria Viruses Detected in Myanmar during the COVID-19 Pandemic in 2021. Viruses.

[13] Foley D.A., Sikazwe C.T., Minney-Smith C.A., Ernst T., Moore H.C., Nicol M.P., Smith D.W., Levy A., Blyth C.C. (2022). An Unusual Resurgence of Human Metapneumovirus in Western Australia Following the Reduction of Non-Pharmaceutical Interventions to Prevent SARS-CoV-2 Transmission. Viruses.

[14] Wagatsuma K., Koolhof I.S., Saito R. (2022). Was the Reduction in Seasonal Influenza Transmission during 2020 Attributable to Non-Pharmaceutical Interventions to Contain Coronavirus Disease 2019 (COVID-19) in Japan?. Viruses.

[15] World Health Orgnization Press Briefing by the International Team Studying the Origins of the COVID-19 Virus—30 March 2021. https://www.who.int/publications/m/item/press-briefing-by-the-international-team-studying-the-origins-of-the-covid-19-virus-30-march-2021.

[16] World Health Orgnization Independent Assessment of the Origins of SARS-CoV-2. 27 June 2025. https://www.who.int/publications/m/item/independent-assessment-of-the-origins-of-sars-cov-2-from-the-scientific-advisory-group-for-the-origins-of-novel-pathogens.

[17] Padykula I., Damodaran L., Young K.T., Krunkosky M., Griffin E.F., North J.F., Neasham P.J., Pliasas V.C., Siepker C.L., Stanton J.B. (2024). Pandemic Risk Assessment for Swine Influenza A Virus in Comparative In Vitro and In Vivo Models. Viruses.

[18] AbuBakar U., Amrani L., Kamarulzaman F.A., Karsani S.A., Hassandarvish P., Khairat J.E. (2023). Avian Influenza Virus Tropism in Humans. Viruses.

[19] Liu Q., Zeng H., Wu X., Yang X., Wang G. (2023). Global Prevalence and Hemagglutinin Evolution of H7N9 Avian Influenza Viruses from 2013 to 2022. Viruses.

[20] Guo X., Zhou Y., Yan H., An Q., Liang C., Liu L., Qian J. (2024). Molecular Markers and Mechanisms of Influenza A Virus Cross-Species Transmission and New Host Adaptation. Viruses.

[21] Foley D.A., Minney-Smith C.A., Lee W.H., Oakes D.B., Hazelton B., Ford T.J., Wadia U., Sikazwe C., Moore H.C., Nicol M.P. (2023). Respiratory Syncytial Virus Reinfections in Children in Western Australia. Viruses.

[22] Wagatsuma K., Koolhof I.S., Saito R. (2023). Nonlinear and Multidelayed Effects of Meteorological Drivers on Human Respiratory Syncytial Virus Infection in Japan. Viruses.

[23] Chow V.T.K., Tay D.J.W., Chen M.I.C., Tang J.W., Milton D.K., Tham K.W. (2023). Influenza A and B Viruses in Fine Aerosols of Exhaled Breath Samples from Patients in Tropical Singapore. Viruses.

[24] del-Puerto F., Rojas L.E., Díaz Acosta C.C., Franco L.X., Cardozo F., Galeano M.E., Valenzuela A., Rojas A., Martínez M., Ayala-Lugo A. (2023). The Experience of Testing for Coronavirus Disease (COVID-19) at a Single Diagnostic Center in Paraguay before the Introduction of Vaccination. Viruses.

[25] Tambe L.A.M., Mathobo P., Munzhedzi M., Bessong P.O., Mavhandu-Ramarumo L.G. (2023). Prevalence and Molecular Epidemiology of Human Coronaviruses in Africa Prior to the SARS-CoV-2 Outbreak: A Systematic Review. Viruses.

[26] Zabidi N.Z., Liew H.L., Farouk I.A., Puniyamurti A., Yip A.J.W., Wijesinghe V.N., Low Z.Y., Tang J.W., Chow V.T.K., Lal S.K. (2023). Evolution of SARS-CoV-2 Variants: Implications on Immune Escape, Vaccination, Therapeutic and Diagnostic Strategies. Viruses.

[27] World Health Orgnization Severe Acute Hepatitis of Unknown Origin in Children—Multicountry. 23 April 2022. https://www.who.int/emergencies/disease-outbreak-news/item/2022-DON376.

[28] Chon I., Win S.M.K., Phyu W.W., Saito R., Kyaw Y., Win N.C., Lasham D.J., Tin H.H., Tamura T., Otoguro T. (2024). Whole-Genome Analysis of the Influenza A(H1N1)pdm09 Viruses Isolated from Influenza-like Illness Outpatients in Myanmar and Community-Acquired Oseltamivir-Resistant Strains Present from 2015 to 2019. Viruses.

[29] Escuyer K.L., Gowie D.L., St George K. (2024). Influenza Virus Surveillance from the 1918 Influenza Pandemic to the 2020 Coronavirus Pandemic in New York State, USA. Viruses.

[30] Jugulete G., Olariu M.C., Stanescu R., Luminos M.L., Pacurar D., Pavelescu C., Merișescu M.M. (2024). The Clinical Effectiveness and Tolerability of Oseltamivir in Unvaccinated Pediatric Influenza Patients during Two Influenza Seasons after the COVID-19 Pandemic: The Impact of Comorbidities on Hospitalization for Influenza in Children. Viruses.

[31] Yoshioka S., Phyu W.W., Wagatsuma K., Nagai T., Sano Y., Taniguchi K., Nagata N., Tomimoto K., Sato I., Kaji H. (2023). Molecular Epidemiology of Respiratory Syncytial Virus during 2019–2022 and Surviving Genotypes after the COVID-19 Pandemic in Japan. Viruses.

[32] Cho S.J., Kim S.H., Mun J., Yun J.E., Park S., Park J., Lee Y.U., Park J.S., Yun H., Lee C.M. (2024). Impact of COVID-19 Pandemic Restrictions on Respiratory Virus Patterns: Insights from RSV Surveillance in Gwangju, South Korea. Viruses.

[33] Foley D.A., Minney-Smith C.A., Tjea A., Nicol M.P., Levy A., Moore H.C., Blyth C.C. (2024). The Changing Detection Rate of Respiratory Syncytial Virus in Adults in Western Australia between 2017 and 2023. Viruses.

[34] Davids M., Johnstone S., Mendes A., Brecht G., Avenant T., du Plessis N., de Villiers M., Page N., Venter M. (2024). Changes in Prevalence and Seasonality of Pathogens Identified in Acute Respiratory Tract Infections in Hospitalised Individuals in Rural and Urban Settings in South Africa; 2018–2022. Viruses.

[35] Di Maio V.C., Scutari R., Forqué L., Colagrossi L., Coltella L., Ranno S., Linardos G., Gentile L., Galeno E., Vittucci A.C. (2024). Presence and Significance of Multiple Respiratory Viral Infections in Children Admitted to a Tertiary Pediatric Hospital in Italy. Viruses.

[36] Reddy B., Simane A., Mthiyane H., Mashishi B., Mbenenge N., Treurnicht F.K. (2024). Prevalence and Seasonal Patterns of 16 Common Viral Respiratory Pathogens during the COVID-19 Pandemic in Gauteng Province, South Africa, 2020–2021. Viruses.

